# Do Weight trajectories influence diabetes control? A prospective study in Switzerland (CoLaus study)

**DOI:** 10.1016/j.pmedr.2021.101473

**Published:** 2021-06-27

**Authors:** Pauline Ducraux, Gérard Waeber, Pedro Marques-Vidal

**Affiliations:** Department of Medicine, Internal Medicine, Lausanne University Hospital and University of Lausanne, 46 rue du Bugnon, 1011 Lausanne, Switzerland

**Keywords:** Diabetes, Diabetes management, Weight, Weight variability, Waist, Epidemiology

## Abstract

**Objective:**

Identify anthropometric trajectories among subjects with type 2 diabetes mellitus (T2DM), and associate them with glycaemic control.

**Methods:**

Prospective study including 268 community-dwelling participants with T2DM (34% women, mean age 68.7 ± 8.9 years) followed for 10.7 years (range: 8.8–13.6 years). T2DM control was considered for 1) fasting plasma glucose (FPG) < 7.0 mmol/L, or 2) HbA_1_c < 7.0% (53 nmol/mol). Changes in weight or waist and weight variability were considered.

**Results:**

One half (FPG) and one third (HbA_1_c) of participants presented with uncontrolled T2DM. Half of the participants presented with obesity and 75% with abdominal obesity. During follow-up, half of the participants maintained their weight, 25% gained > 5 kg, and 25% lost < 5 kg; almost half increased their waist by > 5 cm. Using FPG as criterion, participants who lost > 5 cm waist were more likely to be controlled: multivariable-adjusted odds ratio (OR) and 95% confidence interval (CI): 3.10 (1.23–7.78). Participants with controlled T2DM also presented with a higher weight variability: multivariable adjusted mean ± standard error 4.8 ± 0.3 vs. 3.9 ± 0.3 kg, p = 0.028. Using HbA_1_c as criterion, participants who lost > 5 kg were less likely to be controlled: OR and (95% CI): 0.35 (0.18–0.66). Similar findings were obtained when restricting the analysis to participants who were diabetic throughout the whole study period.

**Conclusion:**

In a Swiss community-based sample of participants with T2DM, T2DM control rates could be implemented. Neither weight nor waist variability was significantly and consistently associated with T2DM control.

## 1 Introduction

Weight gain is closely associated with incidence of type 2 diabetes mellitus (T2DM) (Kataja-Tuomola et al., 2010, Kodama et al., 2014, Wannamethee et al., 2005, Zhang et al., 2017, Zheng et al., 2017). The effect of weight gain occurs irrespective of baseline (de Mutsert et al., 2014) or attained (Kaneto et al., 2013) weight status. Conversely, weight loss is associated with a lower incidence of T2DM (Wannamethee et al., 2005), namely among obese subjects (Robson et al., 2018). Recently, several studies have suggested that body weight variability might also be a risk factor for T2DM (Oh et al., 2019, Park et al., 2019) independently of body mass index (BMI), although this statement has been challenged (Zhang et al., 2017).

Among subjects with T2DM, weight-loss trajectories are associated with better glycaemic control (Feldstein et al., 2008), lower cardiovascular disease (CVD) risk (Look Ahead Research Group et al., 2016) and healthcare costs (Mukherjee et al., 2016). Conversely, weight increase was associated with lower glycaemic control (Vistisen et al., 2014), increased cardiovascular risk (Eeg-Olofsson et al., 2009) and mortality (Bodegard et al., 2013, Kim et al., 2019). Data from a randomized controlled trial on bariatric surgery suggest that the benefit is proportional to the magnitude of weight loss occurring the first year after the surgery (Zhou et al., 2019). Still, a cohort study of 8′486 primary care patients with newly diagnosed T2DM found no benefit of weight loss regarding cardiovascular mortality (Bodegard et al., 2013), and another study reported an increase in overall mortality among subjects with T2DM who lost weight compared to those who gained or maintained weight (Doehner et al., 2012). A *meta*-analysis found little if no effect of weight loss on glycaemic control, although most weight changes reported were small (<5% of initial weight) (Franz et al., 2015). Finally, several studies suggested that both body weight increase and variability are associated with increased cardiovascular risk (Bangalore et al., 2018, Yeboah et al., 2019). Whether body weight variability is also associated with glycaemic control has not been assessed.

Hence, our objective was to associate weight trajectories and weight variability with glycaemic control in subjects with T2DM. We hypothesized that subjects with T2DM who lost weight and/or waist will achieve a better glycaemic control.

Participants and methods

### 1.1 Participants

We used data from the CoLaus study, a prospective, population-based study aimed at assessing the prevalence and determinants of cardiovascular disease. The methodology of the CoLaus study has been reported elsewhere (Firmann et al., 2008). Briefly, a single-step random sampling of the population aged 35 to 75 years at baseline living in the city of Lausanne (Switzerland) was conducted and a baseline sample of 6′733 participants (participation rate 41%) was obtained. The baseline survey was conducted between June 2003 and May 2006; the first follow-up was conducted between April 2009 and September 2012 (median follow-up time 5.4 years, range 4.5–8.8 years), and the second follow-up was conducted between May 2014 and April 2017 (median follow-up time 10.7 years, range 8.8–13.6 years).

### 1.2 Diabetes treatment and control

Participants reported all medicines (either prescribed by a doctor or self-prescribed) taken during the last month. Antidiabetic treatment was defined as any oral antidiabetic or insulin medication according to the WHO anatomical therapeutic chemical classification; biguanides, insulin, dipeptyl peptidase-4 inhibitors (DPP4), glucagon-like peptide-1 analogues (GLP1) and sodium-glucose co-transporter 2 inhibitors (SGLT2).

Blood was collected in the morning after an overnight fast. Biological assays were performed by the CHUV Clinical Laboratory on fresh blood samples within 2 h of blood collection. Measurements were performed on a Cobas 8000 (Roche Diagnostics, Basel, Switzerland). Glucose levels were measured by the glucose hexokinase method, with maximum inter and intra-batch coefficients of variation of 1.6% and 0.8%, respectively. Glycated haemoglobin (HbA_1_c) levels were measured by high performance liquid chromatography using Bio-Rad D-10TM system, with measurement range 3.8% to 18.5% (18 to 179 mmol/mol). T2DM control was defined by 1) a fasting plasma glucose < 7.0 mmol/L; 2) a HbA_1_c < 7.0% (53 nmol/mol) irrespective of age (Cosentino et al., 2019) and 3) a HbA_1_c < 7.0% (53 nmol/mol) for participants aged < 65 years and HbA_1_c < 7.5% (58 nmol/mol) for participants aged ≥ 65 years (American Diabetes Association, 2020).

### 1.3 Weight change and variability

The same protocol for anthropometric measurements was applied in all surveys. Body weight and height were measured with participants barefoot and in light indoor clothes. Body weight was measured in kilograms to the nearest 100 g using a Seca® scale (Hamburg, Germany). Height was measured to the nearest 5 mm using a Seca® (Hamburg, Germany) height gauge. Waist circumference was measured mid-way between the lowest rib and the iliac crest using a non-stretchable tape and the average of two measurements was taken. Abdominal obesity was defined as a waist circumference > 102 cm (men) or > 88 cm (women).

Weight change was defined as the difference between the first and the last visit and three metrics were used: 1) as a continuous variable; 2) categorized into losers (loss > 5% of initial weight), gainers (>5% of initial weight) and maintainers (other) (Park et al., 2019, Franz et al., 2015), and 3) using a threshold of 5 kg weight change (Marques-Vidal et al., 2018). Weight variability was assessed using the average successive variability (ASV), defined as the absolute difference between successive weight measurements:

$$\mathrm{ASV}=\frac{\sum_{i=n}^{i=1}\left|W_{t_{i}}-W_{t_{i+1}}\right|}{\left(n-1\right)}$$

where W*_ti_* = weight at time *i* and n = number of measurements. ASV was used as a continuous variable. Waist change was defined as the difference between the first and the last visit and two metrics were used: 1) as a continuous variable; 2) categorized into losers (loss > 5 cm), gainers (increase > 5 cm) and maintainers (other).

The variability independent of the mean (VIM) was computed for weight and waist according to (Echouffo-Tcheugui et al., 2019) as follows:

$$\mathrm{VIM}=100\times \frac{\mathrm{SD}}{{\mathrm{mean}}^{\beta }}$$

where β is the regression coefficient base on natural logarithm of SD on natural logarithm of the mean.

### 1.4 Covariates

Other covariates were collected using self-filled questionnaires: gender; age; smoking status (never, former, current); presence of a diet (yes/no); marital status (living alone/living in couple); educational level (mandatory, apprenticeship, high school and university) and alcohol consumption (yes/no). Antihypertensive and hypolipidaemic drug treatment were assessed from the list of medicines taken by the participants (self-reported).

Blood pressure (BP) was measured thrice using an Omron® HEM-907 automated oscillometric sphygmomanometer after at least a 10-minute rest in a seated position, and the average of the last two measurements was used. Hypertension was defined by a systolic BP (SBP) ≥ 130 mm Hg or a diastolic BP (DBP) ≥ 80 mm Hg or presence of antihypertensive drug treatment.

Plasma total cholesterol levels were assessed by cholesterol oxidase phenol 4-aminoantipyrine peroxidase (CHOD-PAP), with maximum inter and intra-batch coefficients of variation of 1.6% and 1.7%, respectively. Plasma HDL-cholesterol levels were assessed by CHOD-PAP + Polyethylene glycol + cyclodextrin with maximum inter and intra-batch coefficients of variation of 3.6% and 0.9%, respectively. Triglyceride levels were assessed by glycerol phosphate oxidase-PAP with maximum inter and intra-batch coefficients of variation of 2.9% and 1.5%, respectively. LDL cholesterol levels were assessed using the Friedewald formula. Dyslipidaemia was defined by 1) a LDL cholesterol level ≥ 1.8 mmol/l or presence of hypolipidaemic drug treatment, or 2) a non-HDL cholesterol level ≥ 2.6 mmol/L or presence of hypolipidaemic drug treatment.

### 1.5 Inclusion and exclusion criteria

Participants were included if they presented with diabetes mellitus at the second follow-up, irrespective of their status at baseline and at the first follow-up. Participants were excluded if they 1) reported to have type 1 diabetes mellitus; 2) did not benefit from antidiabetic drug treatment at the second follow-up; 3) had less than two weight measurements, and 4) had missing data for any covariate at the second follow-up (age, gender, smoking status, alcohol consumption or marital status).

### 1.6 Statistical analysis

Statistical analyses were performed using Stata version 16.1 for windows® (Stata Corp, College Station, Texas, USA). Descriptive results were expressed as number of participants (percentage) for categorical variables and as average ± standard deviation (SD) or median [interquartile range] for continuous variables. Bivariate analyses were performed using chi-square or Fisher’s exact test for categorical variables and Student’s *t*-test or nonparametric Kruskal-Wallis test for continuous variables. Multivariate analysis was performed using logistic regression and the results were expressed as Odds ratio (OR) and 95% confidence interval (CI). Statistical significance was assessed for p < 0.05.

Three sensitivity analyses were conducted: 1) restricting the analysis to participants with T2DM (with or without treatment) during the whole study period (i.e. from baseline to the second follow-up); 2) restricting the analysis to participants treated for T2DM during the whole study period and 3) Categorizing glycemic control at both follow-ups as “No-No”, “No-Yes”, “Yes-No” and “Yes-Yes”.

### Ethical statement

The institutional Ethics Committee of the University of Lausanne, which afterwards became the Ethics Commission of Canton Vaud (www.cer-vd.ch) approved the baseline CoLaus study. The approval was renewed for the first and the second follow-ups. The study was performed in agreement with the Helsinki declaration and its former amendments, and in accordance with the applicable Swiss legislation. All participants gave their signed informed consent before entering the study.

## 2 Results

### 2.1 Characteristics of participants

**Fig. 1:**
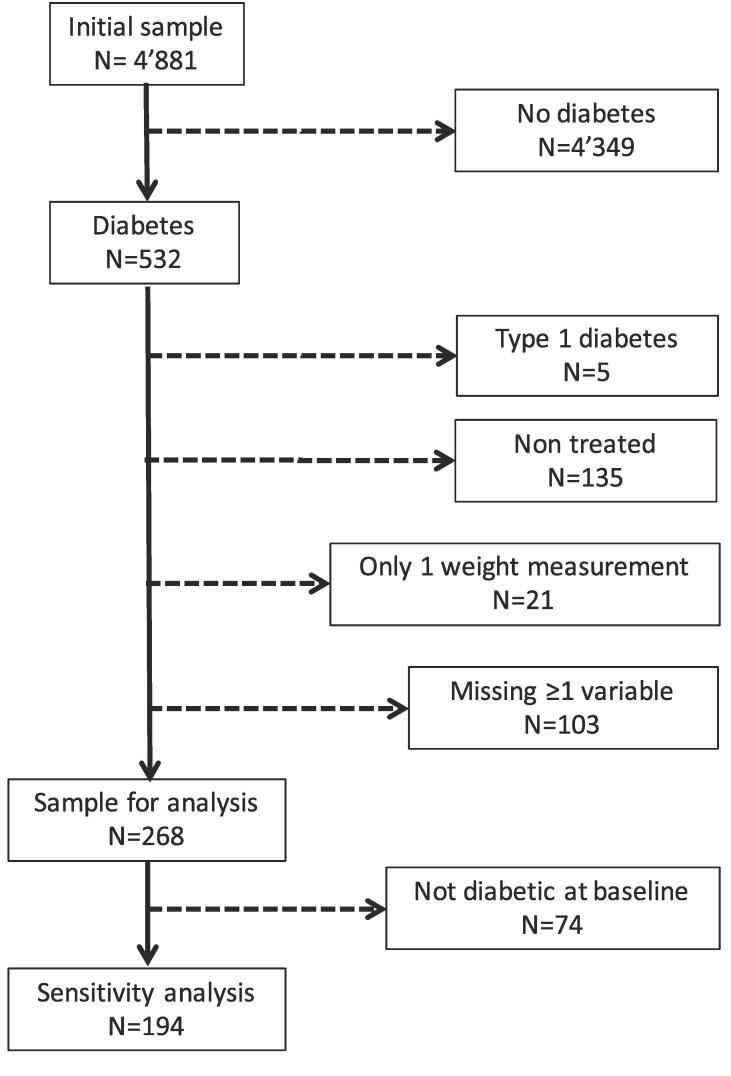
Selection procedure.

Out of the 4′881 participants, 4′349 were considered as non-eligible because they had no diabetes. Of the remaining 532 participants with diabetes, 268 (50.4%) were included in the analysis. The reasons for exclusion are indicated in Fig. 1 and the characteristics of the included and the excluded participants are provided in Supplementary Table 1. Excluded participants were less likely to be on a diet, had higher DBP levels and higher total, LDL and non-HDL cholesterol levels, albeit no difference in dyslipidaemia was found. Median and IQR for the delay between the baseline and the second FU was 10.7 [10.6–10.9], and corresponding 5^th^ and 95^th^ percentiles were 10.4 and 11.7 years.

### 2.2 trajectories according to diabetes control using plasma glucose

The clinical characteristics of participants with controlled and uncontrolled T2DM according to fasting plasma glucose at second follow-up are shown on Table 1. Half of the participants presented with uncontrolled T2DM; the majority were hypertensive and nearly all had dyslipidaemia. Participants with uncontrolled T2DM were younger, had higher SBP levels and higher total, LDL and non-HDL cholesterol levels than participants with controlled T2DM, while no significant differences were found for the other characteristics (Table 1). Four out of five patients (81.3%) were on biguanides, 18.4% on insulin, 6.4% on DDP4, 0.8% on GLP1 and 3.8% of SGLT2; no differences were found regarding drug treatment between controlled and uncontrolled T2DM (not shown).

**Table 1:** Characteristics of the participants with type 2 diabetes mellitus, according to diabetes control status (no/yes) at the second follow-up (2014–2017) of the CoLaus study, Lausanne, Switzerland.

	**Using FPG levels (n = 267)**			**HbA_1_c level < 7.0% (n = 266)**			**Age-dependent HbA_1_c levels (n = 266)**		
	**No**	**Yes**	**p-value**	**No**	**Yes**	**p-value**	**No**	**Yes**	**p-value**
Number (%)	130 (48.7)	137 (51.3)		94 (35.3)	172 (64.7)		69 (25.9)	197 (74.1)	
Age (years)	66.9 ± 8.8	70.3 ± 8.6	0.001	67.7 ± 9.1	69.1 ± 8.7	0.218	65.5 ± 9.5	69.7 ± 8.4	<0.001
Women (%)	43 (33.1)	48 (35.0)	0.736	31 (33.0)	60 (34.9)	0.754	20 (29.0)	71 (36.0)	0.288
Marital status (%)			0.459			0.554			0.904
Alone	53 (40.8)	62 (45.3)		38 (40.4)	76 (44.2)		30 (43.5)	84 (42.6)	
In couple	77 (59.2)	75 (54.7)		56 (59.6)	96 (55.8)		39 (56.5)	113 (57.4)	
Educational level (%)			0.544			0.302			0.431
High	14 (10.8)	17 (12.5)		13 (13.8)	18 (10.5)		9 (13.0)	22 (11.2)	
Middle	33 (25.4)	27 (19.9)		25 (26.6)	35 (20.5)		19 (27.5)	41 (20.9)	
Low	83 (63.9)	92 (67.7)		56 (59.6)	118 (69)		41 (59.4)	133 (67.9)	
Smoking status (%)			0.064			0.897			0.717
Never	33 (25.4)	53 (38.7)		29 (30.9)	57 (33.1)		20 (29.0)	66 (33.5)	
Former	70 (53.9)	59 (43.1)		47 (50.0)	81 (47.1)		36 (52.2)	92 (46.7)	
Current	27 (20.8)	25 (18.3)		18 (19.2)	34 (19.8)		13 (18.8)	39 (19.8)	
Alcohol drinker (%)	86 (66.2)	79 (57.7)	0.154	59 (62.8)	106 (61.6)	0.855	46 (66.7)	119 (60.4)	0.356
On a diet (%)	59 (45.4)	61 (44.5)	0.888	45 (47.9)	74 (43.0)	0.447	34 (49.3)	85 (43.2)	0.378
Blood pressure (mm Hg)									
Systolic	136 ± 18	131 ± 17	0.026	134 ± 17	133 ± 19	0.759	133 ± 17	134 ± 18	0.888
Diastolic	77 ± 10	76 ± 11	0.429	77 ± 10	76 ± 10	0.835	78 ± 10	76 ± 10	0.252
Hypertension (%)	117 (90.0)	125 (91.2)	0.728	85 (90.4)	156 (90.7)	0.942	64 (92.8)	177 (89.9)	0.477
Cholesterol levels (mmol/L)									
Total	4.78 ± 1.03	4.34 ± 1.02	<0.001	4.69 ± 1.10	4.49 ± 1.02	0.154	4.69 ± 1.06	4.51 ± 1.04	0.221
HDL	1.28 ± 0.39	1.30 ± 0.33	0.669	1.29 ± 0.39	1.30 ± 0.35	0.804	1.23 ± 0.34	1.32 ± 0.37	0.112
LDL	2.64 ± 0.97	2.39 ± 0.87	0.026	2.56 ± 1.02	2.49 ± 0.88	0.531	2.58 ± 0.99	2.49 ± 0.91	0.540
Non-HDL	3.50 ± 1.06	3.04 ± 1.00	<0.001	3.40 ± 1.14	3.19 ± 0.99	0.132	3.46 ± 1.10	3.20 ± 1.03	0.076
Dyslipidemia (%)	129 (1 0 0)	134 (97.8)	0.248 §	93 (1 0 0)	169 (98.3)	0.554 §	68 (1 0 0)	194 (98.5)	0.306 §

FPG, fasting plasma glucose. Results are expressed as number of participants (percentage) for categorical variables and as average ± standard deviation for continuous variables. Between group comparisons performed using chi-square or Fisher’s exact test (§) for categorical variables and student’s *t*-test for continuous variables. One participant had missing data for dyslipidemia.

The weight trajectories of participants with controlled and uncontrolled T2DM according to fasting plasma glucose at second follow-up are shown on tables 2 (bivariate) and **3** (multivariable). Half of the participants maintained their weight, one quarter gained > 5 kg, and one quarter lost < 5 kg.; almost half of the participants increased their waist by > 5 cm. On bivariate analysis, participants with controlled T2DM lost > 5 cm waist more frequently than participants with uncontrolled T2DM; this finding was confirmed on multivariate analysis, where participants who lost > 5 cm waist had an over three-fold higher likelihood of being controlled (Table 3). Participants with controlled T2DM also had a higher ASV than participants with uncontrolled T2DM after multivariate adjustment (Table 3). No significant differences were found for all other anthropometric markers.

**Table 2:** Bivariate analysis of weight parameters of the participants with type 2 diabetes mellitus, according to diabetes control status at the second follow-up (2014–2017) of the CoLaus study, Lausanne, Switzerland.

	**Using FPG levels (n = 267)**			**HbA_1_c level < 7.0% (n = 266)**			**Age-dependent HbA_1_c levels (n = 266)**		
	**No**	**Yes**	**p-value**	**No**	**Yes**	**p-value**	**No**	**Yes**	**p-value**
Number	130 (48.7)	137 (51.3)		94 (35.3)	172 (64.7)		69 (25.9)	197 (74.1)	
**Body mass index data**									
Body mass index (kg/m^2^)	30.3 ± 4.9	30.4 ± 5.0	0.903	30.2 ± 4.7	30.4 ± 5.0	0.719	30.8 ± 4.6	30.1 ± 5.0	0.343
BMI category (%)			0.980			0.998			0.464
Normal	17 (13.0)	17 (12.4)		12 (12.8)	22 (12.8)		6 (8.7)	28 (14.2)	
Overweight	50 (38.5)	54 (39.4)		37 (39.3)	67 (39.0)		27 (39.1)	77 (39.1)	
Obese	63 (48.5)	66 (48.2)		45 (47.9)	83 (48.2)		36 (52.2)	92 (46.7)	
**Weight data**									
Weight change (kg)	0.4 ± 7.3	−0.1 ± 8.9	0.642	−0.6 ± 8.0	0.5 ± 8.2	0.322	0.3 ± 8.3	0 ± 8.1	0.825
Weight change categories (%)			0.927			0.011			0.569
Lost > 5 kg	32 (24.6)	31 (22.6)		32 (34.0)	31 (18.0)		19 (27.5)	44 (22.3)	
Maintained	68 (52.3)	73 (53.3)		41 (43.6)	100 (58.1)		33 (47.8)	108 (54.8)	
Gained > 5 kg	30 (23.1)	33 (24.1)		21 (22.3)	41 (23.8)		17 (24.6)	45 (22.8)	
% weight change	0.8 ± 8.5	0.3 ± 10.5	0.712	−0.3 ± 9.2	1.0 ± 9.7	0.307	0.8 ± 9.4	0.4 ± 9.6	0.760
% weight change categories (%)			0.895			0.127			0.882
Lost > 5%	35 (26.9)	35 (25.6)		31 (33.0)	39 (22.7)		18 (26.1)	52 (26.4)	
Maintained	57 (43.9)	64 (46.7)		36 (38.3)	85 (49.4)		30 (43.5)	91 (46.2)	
Gained > 5%	38 (29.2)	38 (27.7)		27 (28.7)	48 (27.9)		21 (30.4)	54 (27.4)	
ASV (kg)	3.4 [2.3–5.1]	4.3 [2.2–6.5]	0.144	3.5 [2.1–5.5]	4.1 [2.3–6.2]	0.686	3.5 [2.1–5.4]	4.0 [2.3–6.2]	0.699
VIM	17.1 [10.7–26.4]	20.4 [10.1–32.5]	0.122	18.3 [10.5–27.5]	17.4 [10.6–29.9]	0.941	17.2 [9.7–26.4]	18.1 [10.7–29.8]	0.491
**Waist data**									
Waist (cm)	106 ± 12	106 ± 13	0.873	105 ± 11	106 ± 13	0.914	107 ± 11	105 ± 13	0.213
Abdominal obesity (%)	93 (71.5)	102 (75.0)	0.523	67 (71.3)	127 (74.3)	0.599	51 (73.9)	143 (73.0)	0.878
Waist change (cm)	3.7 ± 7.0	3.2 ± 11.4	0.695	3.0 ± 7.8	3.6 ± 10.3	0.611	3.5 ± 8.0	3.4 ± 10.0	0.930
Waist change categories (%)			0.034			0.500			0.872
Lost > 5 cm	9 (6.9)	22 (16.2)		12 (12.8)	19 (11.1)		7 (10.1)	24 (12.2)	
Maintained	63 (48.5)	51 (37.5)		44 (46.8)	70 (40.9)		31 (44.9)	83 (42.4)	
Gained > 5 cm	58 (44.6)	63 (46.3)		38 (40.4)	82 (48.0)		31 (44.9)	89 (45.4)	
ASV (cm)	4.5 [2.6–6.8]	5.3 [3.3–8.3]	0.010 §	4.5 [2.8–7.5]	4.9 [3.0–7.5]	0.327 §	4.8 [2.8–8.0]	4.8 [3.0–7.3]	0.909 §
VIM	2.7 [1.8–4.2]	3.5 [2.2–5.4]	0.004 §	3.0 [1.6–4.6]	3.2 [2.1–4.9]	0.208 §	3.2 [1.7–4.7]	3.1 [2.1–4.7]	0.585 §

ASV, average successive variability of weight; BMI, body mass index; FPG, fasting plasma glucose; VIM, variability independent of the mean. Results are expressed as number of participants (percentage) for categorical variables and as average ± standard deviation or as median [interquartile range] for continuous variables. Bivariate analysis performed using chi-square for categorical variables and student’s *t*-test or Kruskal-Wallis test (§) for continuous variables.

**Table 3:** multivariable analysis of weight parameters of the participants with type 2 diabetes mellitus, according to diabetes control status (yes/no) at the second follow-up (2014–2017) of the CoLaus study, Lausanne, Switzerland.

	**Using FPG levels (n = 267)**			**HbA_1_c level < 7.0% (n = 266)**			**Age-dependent HbA_1_c levels (n = 266)**		
	**No**	**Yes**	**p-value**	**No**	**Yes**	**p-value**	**No**	**Yes**	**p-value**
Number	130 (48.7)	137 (51.3)		94 (35.3)	172 (64.7)		69 (25.9)	197 (74.1)	
**Body mass index data**									
Body mass index (kg/m^2^)	30.1 ± 0.4	30.6 ± 0.4	0.371	30.0 ± 0.5	30.4 ± 0.3	0.464	30.2 ± 0.6	30.3 ± 0.3	0.918
BMI category									
Normal		1 (ref)			1 (ref)			1 (ref)	
Overweight		0.96 (0.42–2.19)	0.923		0.95 (0.41–2.19)	0.900		0.64 (0.23–1.81)	0.405
Obese		1.15 (0.50–2.68)	0.739		1.04 (0.44–2.45)	0.925		0.67 (0.24–1.91)	0.453
**Weight data**									
Weight change (kg)	−0.3 ± 0.7	0.4 ± 0.7	0.448	−0.9 ± 0.8	0.6 ± 0.6	0.131	−0.9 ± 0.9	0.4 ± 0.5	0.224
Weight change categories									
Lost > 5 kg		0.74 (0.39–1.40)	0.349		0.35 (0.18–0.66)	0.001		0.57 (0.28–1.17)	0.126
Maintained		1 (ref.)			1 (ref.)			1 (ref)	
Gained > 5 kg		1.32 (0.69–2.55)	0.401		0.90 (0.46–1.77)	0.758		1.10 (0.52–2.32)	0.807
% weight change	0 ± 0.8	1.0 ± 0.8	0.366	−0.7 ± 0.9	1.1 ± 0.7	0.125	−0.6 ± 1.1	0.8 ± 0.6	0.260
% weight change categories									
Lost > 5%		0.67 (0.35–1.28)	0.223		0.45 (0.23–0.86)	0.016		0.27 (1.48–1.17)	0.342
Maintained		1 (ref.)			1 (ref.)			1 (ref)	
Gained > 5%		0.99 (0.53–1.87)	0.987		0.78 (0.41–1.50)	0.457		0.37 (2.10–2.32)	0.502
ASV (kg)	3.9 ± 0.3	4.8 ± 0.3	0.028	4.2 ± 0.3	4.4 ± 0.2	0.553	4.1 ± 0.4	4.4 ± 0.2	0.509
VIM	18.9 ± 1.3	23.9 ± 1.3	0.008	20.7 ± 1.5	21.8 ± 1.1	0.575	20.1 ± 1.8	21.9 ± 1.1	0.392
**Waist data**									
Waist (cm)	105 ± 1	106 ± 1	0.459	105 ± 1	106 ± 1	0.599	106 ± 1	105 ± 1	0.821
Abdominal obesity									
Normal		1 (ref.)			1 (ref)			1 (ref)	
Obese		1.15 (0.62–2.14)	0.653		1.17 (0.62–2.18)	0.631		0.98 (0.48–1.99)	0.957
Waist change (cm)	3.0 ± 0.8	3.8 ± 0.8	0.472	2.6 ± 1.0	3.8 ± 0.7	0.321	2.4 ± 1.2	3.7 ± 0.7	0.316
Waist change categories									
Lost > 5 cm		3.10 (1.23–7.78)	0.016		1.00 (0.43–2.33)	0.999		1.21 (0.45–3.28)	0.709
Maintained		1 (ref.)			1 (ref.)			1 (ref)	
Gained > 5 cm		1.70 (0.97–2.97)	0.063		1.55 (0.88–2.74)	0.127		1.34 (0.72–2.50)	0.361
ASV (cm)	4.8 ± 0.3	6.1 ± 0.3	0.002	5.1 ± 0.3	5.6 ± 0.2	0.313	5.4 ± 0.4	5.4 ± 0.2	0.984
VIM	3.0 ± 0.2	4.1 ± 0.2	<0.001	3.2 ± 0.2	3.7 ± 0.2	0.059	3.3 ± 0.3	3.6 ± 0.1	0.290

ASV, average successive variability of weight; BMI, body mass index; FPG, fasting plasma glucose; VIM, variability independent of the mean. Analysis was performed separately for each anthropometric variable (row), and models are not adjusted for the other row variables. Multivariable analysis for categorical variables was performed using logistic regression and results are expressed as odds ratio (95% confidence interval). Multivariable analysis for continuous variables was performed using analysis of variance and results are expressed as adjusted average ± standard error. Multivariable analysis adjusted for gender, age (continuous), educational level (mandatory, apprenticeship, high school and university), marital status (alone/couple), smoking status (never, former, current), alcohol consumption (yes/no), presence of a diet (yes/no), antihypertensive drug treatment (yes/no) and hypolipidemic drug treatment (yes/no).

Sensitivity analyses restricted to participants with T2DM (with or without treatment) during the whole study period (**Supplementary** Tables 2 and 3), or to participants treated for T2DM during the whole study period (Supplementary **tables 4 and 5**) led to similar findings, i.e. lack of consistent association of levels or changes in anthropometric markers with diabetes control. Of the 81 participants treated for T2DM during the whole study period, 32 (39.5%) were not controlled. Further adjustment on antidiabetic drug categories showed that participants who lost > 5 cm waist had three-fold higher likelihood of being controlled, while ASV was significantly higher in participants with controlled T2DM (**Supplementary table 6**). No differences regarding weight gain were found after categorizing glycemic control as “No-No”, “No-Yes”, “Yes-No” and “Yes-Yes” (**Supplementary** Fig. 1, Fisher’s exact test p = 0.629)

### 2.3 Weight trajectories according to diabetes control using glycated haemoglobin

The clinical characteristics of controlled and uncontrolled T2DM participants according to a single Hba_1_c level at second follow-up are shown on table 1. One third of participants presented with uncontrolled T2DM, a majority presented with hypertension and nearly all had dyslipidaemia, while no significant difference was found for the other characteristics (Table 1). Using age-dependent Hba_1_c levels to define T2DM control showed 74% control rate; controlled participants were older, while no significant difference was found for the other characteristics (Table 1).

The weight trajectories of participants with controlled and uncontrolled T2DM according to a single Hba_1_c level at second follow-up are shown on tables 2 (bivariate) and **3** (multivariable). On bivariate analysis, participants with uncontrolled T2DM lost > 5 kg more frequently than participants with controlled T2DM; this finding was confirmed on multivariate analysis, where participants who had lost > 5 kg were less likely to be controlled (Table 3). No significant differences were found for all other anthropometric markers (Table 2, Table 3). Using age-dependent Hba_1_c levels to define T2DM control led to non-significant differences regarding all anthropometric markers studied (Table 2, Table 3).

Sensitivity analyses restricted to participants with T2DM (with or without treatment) during the whole study period (**Supplementary** Table 2, Table 3), or to participants treated for T2DM during the whole study period (**Supplementary tables 4 and 5**) led to similar findings, i.e. lack of consistent association of levels or changes in anthropometric markers with diabetes control. Further adjustment on antidiabetic drug categories showed that participants who lost > 5 kg were less likely to be controlled and that participants with uncontrolled T2DM lost weight more frequently than participants with controlled T2DM (**supplementary table 6**). Using age-dependent Hba_1_c levels to define T2DM control led to similar findings (**supplementary** Table 2, Table 3).

## 3 Discussion

In this population-based study, over one third of participants with T2DM had their disease uncontrolled, and only a quarter managed to lose weight. Contrary to our initial hypothesis, participants with uncontrolled T2DM tended to lose weight more frequently than participants with controlled T2DM. Overall, neither weight or waist status, nor its evolution were significantly and consistently associated with T2DM control.

### 3.1 Diabetes control

Diabetes control can be monitored on the short-term by fasting plasma glucose levels, while HbA_1_c levels should be used for the long-term monitoring (Cosentino et al., 2019). In this study, at least one quarter of participants with T2DM were uncontrolled, a far from optimal rate. Still, our findings are slightly better than other studies: 38% of uncontrolled subjects in Bulgaria, Croatia, Poland, Romania and the UK (Gyberg et al., 2015) and 30 to 50% in the USA (Feldstein et al., 2008), with even higher rates in other countries: 60% in Jordan (Al-Eitan et al., 2016) and 70% in China (Chen et al., 2015). The relatively low control rates in our study could be explained by several factors. Firstly, a low adherence to anti-diabetic treatment, although this hypothesis has been challenged (Gyberg et al., 2015, Michiels et al., 2019). Secondly, participants with T2DM might have a suboptimal knowledge regarding the management of their disease (Chen et al., 2015), while the implementation of educational tools improves glycaemic control (Michiels et al., 2019, Defeudis et al., 2018). As no data regarding T2DM knowledge was collected, this hypothesis should be further assessed. Thirdly, doctors might have difficulty in selecting which drugs to prescribe, particularly the new classes of anti-diabetic drugs (Jornayvaz and Gariani, 2020) and in the presence of multiple CVD risk factors. Lastly, doctors and patients might have differing opinions regarding the disease and its management: doctors prioritize HbA_1_c, diabetic complications and hypoglycaemia, while patients focus on quality of life (Puder et al., 2006, Brod et al., 2016). Restricting the analysis to participants with T2DM during the whole study period showed that diabetes management does not improve with time, with even lower results regarding glycaemic control. Irrespective of the possible causes, our results indicate that, management of T2DM can still be implemented in the Swiss population, namely by using the new antidiabetic drugs, which also promote weight loss.

### 3.2 trajectories according to diabetes control

Half of the participants were obese as assessed by BMI, and only one out of seven presented with normal weight. During the 10.7-year follow-up, half of the participants maintained their weight, one quarter gained > 5 kg, and one quarter lost < 5 kg. Those findings are close to a Swedish study conducted among 8′486 T2DM patients, of whom 53.4% maintained their weight, 14.4% increased and 32.2% decreased (Bodegard et al., 2013). Both obesity and weight gain have been associated with an increase in CVD among subjects with T2DM (Eeg-Olofsson et al., 2009), although a recent study found the opposite association (Doehner et al., 2020). Current guidelines recommend weight loss for T2DM patients (Cosentino et al., 2019), but weight loss is seldom achieved in practice; an Iranian study suggested that lower educational and financial status were major obstacles to weight loss (Jalilian et al., 2019). Still, no differences in social characteristics were found between weight change groups in our study. Another possibility is that subjects with T2DM do not consider weight loss as a way to control their diabetes (Jalilian et al., 2019).

A seemingly paradoxical finding was that subjects with uncontrolled T2DM tended to lose weight more frequently than subjects with controlled T2DM. This finding could be explained by two hypotheses: either uncontrolled T2DM leads to weight loss (Riediger et al., 2017), or weight loss could be due to a reinforcement of medical care, with diet or medication ordered by the doctor following low control status. The latter seems to be more likely in our study, as weight loss was associated with increased use of antidiabetic drugs.

Almost three quarters of participants presented with abdominal obesity, and only one out of six managed to reduce WC by > 5 cm, while almost half of participants increased their WC by > 5 cm. Our findings are in agreement with other studies, where increasing WC was found among subjects with T2DM (De Backer et al., 2016). Increased WC has been shown to be a major determinant of T2DM incidence (Hu et al., 2019, Jeon et al., 2019) and control (Hameed and AbdulQahar, 2019, Mamo et al., 2019). Hence, decreasing WC and thus abdominal obesity could favour T2DM control. Indeed, in our study, participants who lost > 5 cm WC during follow-up were three times more likely to be controlled, although this association was not observed with HbA_1_c. A similar finding was reported in a Japanese study, where obese participants with prediabetes who lost WC returned to normoglycaemia (Hu et al., 2019). Overall, our results suggest that decreasing WC might be beneficial for T2DM management.

Participants with controlled T2DM tended to present with higher ASV levels than participants with uncontrolled T2DM. Our findings somewhat contradict two previous studies, where body weight variability was associated with increased CVD risk (Bangalore et al., 2018, Yeboah et al., 2019). A possible explanation is that our sample size is underpowered to detect small differences in ASV, or that the number of weight measurements available (3 vs. 12 in the study of Bangalore et al. (Bangalore et al., 2018) was too small to adequately assess ASV, although it is the same as in the study of Yeboah et al (Yeboah et al., 2019). Based on our findings, the role of ASV on T2DM control should be further examined.

### 3.3 Implications for clinical practice

Our results highlight the need for better follow-up and possibly a more aggressive management of subjects with T2DM in Switzerland. Whenever possible, shifting to weight-reducing antidiabetic drugs such as glucagon-like peptide 1 (GLP-1) agonists could also be considered. Weight loss strategies should also be implemented, considering that strict dietetary control during 8 weeks proved to be efficient for weight loss and insulin resistance (Christensen et al., 2018, Hansen et al., 2018) and that intensive lifestyle intervention such as LookAHEAD study implemented for a year had short and long term gains on glycemic control, fitness and CVD risk factors (Look Ahead Research Group et al., 2007, Look Ahead Research Group and Wing, 2010, Look Ahead Research Group, 2014).

### 3.4 Strengths and limitations

The major strengths of this study are its prospective setting, the use of two different markers for T2DM control and the large array of obesity markers studied.

This study also has several limitations. Firstly, the study was conducted in a European country, with a high performing and responsive health system (OECD/WHO, 2011). Hence, generalization to other countries with a different health system might not be valid. Still, the associations between weight trajectories and T2DM control are expected to hold true irrespective of the health system considered. Secondly, a sizable fraction of participants with T2DM was excluded, namely those with the highest levels of CVD risk factors. Hence, it is likely that our estimates are biased towards optimism, and that the real prevalence of uncontrolled T2DM and/or the impact of weight trajectories on T2DM control might be higher. No postprandial glucose data was available. Hence, it was not possible to assess the associations between weight or waist markers and possibly undetected diabetes as per FPG. Also, discrepant results were found when using PFG and HbA1c levels. A possible explanation is that the significant associations were due to chance, as the number of comparisons was large and most results pointed towards the lack of association between weight or waist markers and T2DM control. Considering that 12 wt or waist markers were used per definition of T2DM control, then the significance level should have been at most 0.05/12 = 0.004 and no significant association between weight or waist markers and T2DM control would have been found for the whole analysis. Finally, and as indicated above, sample size and the number of weight measurements were small. Hence, it is likely that some associations were not detected.

We conclude that in a Swiss community-based sample of participants with T2DM, T2DM control rates could be implemented. Neither weight nor waist variability was significantly and consistently associated with T2DM control.

Funding

The CoLaus study was and is supported by research grants from GlaxoSmithKline (no grant number), the Faculty of Biology and Medicine of Lausanne (no grant number), and the Swiss National Science Foundation (grants 33CSCO-122661, 32003B_173092, 33CS30-139468 and 33CS30-148401).

Data access

Due to the sensitivity of the data and the lack of consent for online posting, individual data cannot be made accessible. Only metadata will be made available in digital repositories. Metadata requests can also be performed via the study website www.colaus-psycolaus.ch.

## CRediT authorship contribution statement

**Pauline Ducraux:** Investigation, Writing - original draft, Visualization. **Gérard Waeber:** Conceptualization, Supervision, Writing - review & editing. **Pedro Marques-Vidal:** Data curation, Formal analysis, Writing - review & editing.

## Declaration of Competing Interest

The authors declare that they have no known competing financial interests or personal relationships that could have appeared to influence the work reported in this paper.
