# Supplementary material for: Do Weight trajectories influence diabetes control? A prospective study in Switzerland (CoLaus study)
Source: Prev Med Rep. 2021 Jun 27;23:101473. doi: 10.1016/j.pmedr.2021.101473 (PMC8259406; doi:10.1016/j.pmedr.2021.101473)

**SUPPLEMENTARY TABLES**

**Supplementary table 1**: characteristics of the included and the excluded participants with type 2 diabetes mellitus, CoLaus study, Lausanne, Switzerland

|  | **Included** | **Excluded** | **p-value** |
| --- | --- | --- | --- |
| Number | 268 | 264 |  |
| Age (years) | 68.7 ± 8.9 | 67.9 ± 9.7 | 0.336 |
| Women (%) | 91 (34.0) | 106 (40.2) | 0.139 |
| Marital status (%) |  |  | 0.034 |
| Single | 115 (42.9) | 129 (52.2) |  |
| In couple | 153 (57.1) | 118 (47.8) |  |
| Educational level (%) |  |  | 0.878 |
| High | 31 (11.6) | 33 (12.5) |  |
| Middle | 61 (22.9) | 56 (21.2) |  |
| Low | 175 (65.5) | 175 (66.3) |  |
| Smoking status (%) |  |  | 0.719 |
| Never | 87 (32.5) | 71 (35.5) |  |
| Former | 129 (48.1) | 89 (44.5) |  |
| Current | 52 (19.4) | 40 (20.0) |  |
| Body mass index (kg/m^2^) | 30.3 ± 4.9 | 30.1 ± 4.6 | 0.551 |
| BMI category (%) |  |  | 0.125 |
| Normal | 34 (12.7) | 19 (9.0) |  |
| Overweight | 105 (39.2) | 101 (47.9) |  |
| Obese | 129 (48.1) | 91 (43.1) |  |
| Waist (cm) | 106 ± 12 | 104 ± 12 | 0.114 |
| Abdominal obesity (%) | 195 (73.0) | 151 (71.2) | 0.661 |
| On a diet (%) | 121 (45.2) | 73 (27.7) | <0.001 |
| Blood pressure (mm Hg) |  |  |  |
| Systolic | 134 ± 18 | 135 ± 18 | 0.317 |
| Diastolic | 76 ± 10 | 79 ± 11 | 0.012 |
| Hypertension (%) | 243 (90.7) | 222 (89.5) | 0.660 |
| Cholesterol levels (mmol/L) |  |  |  |
| Total | 4.6 ± 1.0 | 5.1 ± 1.2 | <0.001 |
| HDL | 1.3 ± 0.4 | 1.3 ± 0.4 | 0.155 |
| LDL | 2.5 ± 0.9 | 2.9 ± 1.0 | <0.001 |
| Non-HDL | 3.3 ± 1.1 | 3.7 ± 1.2 | <0.001 |
| Dyslipidemia (%) | 264 (98.9) | 237 (99.2) | 1.000 § |

BMI, body mass index. Results are expressed as number of participants (percentage) for categorical variables and as average ± standard deviation for continuous variables. Between group comparisons performed using chi-square or Fisher’s exact test (§) for categorical variables and student’s t-test for continuous variables. In the “excluded” column, numbers might not add to 264 due to missing data.

**Supplementary table 2**: bivariate analysis of weight parameters of the participants with type 2 diabetes mellitus, according to diabetes control status (yes/no) at the second follow-up (2014-2017) of the CoLaus study, Lausanne, Switzerland. Only participants who were diabetic throughout the whole study period.

|  | **Using FPG levels (n=194)** | | | **HbA_1_c level <7.0% (n=193)** | | | **Age-dependent HbA_1_c levels (n=193)** | | |
| --- | --- | --- | --- | --- | --- | --- | --- | --- | --- |
|  | **No** | **Yes** | **p-value** | **No** | **Yes** | **p-value** | **No** | **Yes** | **p-value** |
| Number | 109 (56.2) | 85 (43.8) |  | 75 (38.9) | 118 (61.1) |  | 53 (27.5) | 140 (72.5) |  |
| **Body mass index data** |  |  |  |  |  |  |  |  |  |
| Body mass index (kg/m^2^) | 30.3 ± 4.8 | 29.9 ± 4.9 | 0.583 | 30.1 ± 4.6 | 30.1 ± 4.9 | 0.964 | 30.7 ± 4.8 | 29.9 ± 4.8 | 0.299 |
| BMI category (%) |  |  | 0.969 |  |  | 0.921 |  |  | 0.738 |
| Normal | 13 (11.9) | 11 (12.9) |  | 9 (12.0) | 15 (12.7) |  | 5 (9.4) | 19 (13.6) |  |
| Overweight | 47 (43.1) | 37 (43.5) |  | 34 (45.3) | 50 (42.4) |  | 24 (45.3) | 60 (42.9) |  |
| Obese | 49 (45.0) | 37 (43.5) |  | 32 (42.7) | 53 (44.9) |  | 24 (45.3) | 61 (43.6) |  |
| **Weight data** |  |  |  |  |  |  |  |  |  |
| Weight change (kg) | -0.4 ± 7.2 | -2.9 ± 8.2 | 0.026 | -1.8 ± 7.8 | -1.4 ± 7.6 | 0.740 | -1.1 ± 8.2 | -1.7 ± 7.5 | 0.583 |
| Weight change categories (%) |  |  | 0.500 |  |  | 0.020 |  |  | 0.462 |
| Lost >5 kg | 31 (28.4) | 26 (30.6) |  | 30 (40.0) | 27 (22.9) |  | 18 (34) | 39 (27.9) |  |
| Maintained | 57 (52.3) | 48 (56.5) |  | 32 (42.7) | 73 (61.9) |  | 25 (47.2) | 80 (57.1) |  |
| Gained >5 kg | 21 (19.3) | 11 (12.9) |  | 13 (17.3) | 18 (15.3) |  | 10 (18.9) | 21 (15.0) |  |
| ASV (kg) | 3.4 [2.3 - 5.0] | 4.1 [2.0 - 6.5] | 0.269 § | 3.5 [2.1 - 5.3] | 3.6 [2.2 - 5.8] | 0.791 § | 3.5 [2.0 - 4.8] | 3.5 [2.3 - 5.8] | 0.559 § |
| VIM | 16.9 [10.9-24.9] | 20.4 [9.6-31.1] | 0.268 § | 18.7 [10.8-26.4] | 16.0 [10.6-28.7] | 0.847 § | 17.2 [10.5-24.9] | 17.1 [10.8-28.9] | 0.463 § |
| **Waist data** |  |  |  |  |  |  |  |  |  |
| Waist (cm) | 106 ± 12 | 106 ± 13 | 0.670 | 106 ± 12 | 106 ± 13 | 0.804 | 108 ± 11 | 105 ± 12 | 0.153 |
| Abdominal obesity (%) | 78 (71.6) | 64 (76.2) | 0.469 | 53 (70.7) | 88 (75.2) | 0.486 | 38 (71.7) | 103 (74.1) | 0.736 |
| Waist change (cm) | 3.2 ± 7.1 | 1.2 ± 12.2 | 0.150 | 2.3 ± 7.8 | 2.3 ± 10.8 | 0.984 | 2.7 ± 8.1 | 2.1 ± 10.3 | 0.736 |
| Waist change categories (%) |  |  | 0.086 |  |  | 0.686 |  |  | 0.903 |
| Lost >5 cm | 9 (8.3) | 16 (19.1) |  | 11 (14.7) | 14 (12.0) |  | 6 (11.3) | 19 (13.7) |  |
| Maintained | 54 (49.5) | 37 (44.0) |  | 37 (49.3) | 54 (46.2) |  | 26 (49.1) | 65 (46.8) |  |
| Gained >5 cm | 46 (42.2) | 31 (36.9) |  | 27 (36) | 49 (41.8) |  | 21 (39.6) | 55 (39.6) |  |
| ASV (cm) | 4.5 [2.8 - 6.5] | 4.5 [3.0 - 7.8] | 0.293 § | 4.5 [2.6 - 7.0] | 4.5 [2.8 - 6.6] | 0.819 § | 4.5 [2.6 - 7.6] | 4.4 [2.8 - 6.5] | 0.760 § |
| VIM | 2.6 [1.9 - 4.2] | 3.2 [2.0 - 4.6] | 0.231 § | 2.9 [1.6 - 4.6] | 2.6 [2.0 - 4.1] | 0.831 § | 3.0 [1.6 - 4.7] | 2.6 [2.0 - 4.1] | 0.970 § |

ASV, average successive variability of weight; BMI, body mass index; FPG, fasting plasma glucose; VIM, variability independent of the mean. Results are expressed as number of participants (percentage) for categorical variables and as average ± standard deviation or as median [interquartile range] for continuous variables. Bivariate analysis performed using chi-square for categorical variables and student’s t-test or Kruskal-Wallis test (§) for continuous variables.

**Supplementary table 3**: multivariable analysis of weight parameters of the participants with type 2 diabetes mellitus, according to diabetes control status (yes/no) at the second follow-up (2014-2017) of the CoLaus study, Lausanne, Switzerland. Participants who were diabetic throughout the whole study period.

|  | **Using FPG levels (n=194)** | | | **HbA_1_c level <7.0% (n=193)** | | | **Age-dependent HbA_1_c levels (n=193)** | | |
| --- | --- | --- | --- | --- | --- | --- | --- | --- | --- |
|  | **No** | **Yes** | **p-value** | **No** | **Yes** | **p-value** | **No** | **Yes** | **p-value** |
| Number | 109 (56.2) | 85 (43.8) |  | 75 (38.9) | 118 (61.1) |  | 53 (27.5) | 140 (72.5) |  |
| **Body mass index data** |  |  |  |  |  |  |  |  |  |
| Body mass index (kg/m^2^) | 30.1 ± 0.4 | 30.3 ± 0.5 | 0.778 | 30.0 ± 0.5 | 30.2 ± 0.4 | 0.759 | 30.2 ± 0.6 | 30.1 ± 0.4 | 0.866 |
| BMI category (%) |  |  |  |  |  |  |  |  |  |
| Normal |  | 1 (ref.) |  |  | 1 (ref.) |  |  | 1 (ref.) |  |
| Overweight |  | 0.95 (0.35 - 2.62) | 0.924 |  | 0.84 (0.31 - 2.27) | 0.728 |  | 0.68 (0.20 - 2.31) | 0.537 |
| Obese |  | 1.02 (0.36 - 2.92) | 0.970 |  | 1.02 (0.36 - 2.86) | 0.972 |  | 0.75 (0.21 - 2.63) | 0.654 |
| **Weight data** |  |  |  |  |  |  |  |  |  |
| Weight change (kg) | -0.9 ± 0.7 | -2.3 ± 0.8 | 0.246 | -2.1 ± 0.9 | -1.2 ± 0.7 | 0.470 | -2 ± 1.1 | -1.4 ± 0.6 | 0.608 |
| Weight change categories |  |  |  |  |  |  |  |  |  |
| Lost >5 kg |  | 0.74 (0.36 - 1.54) | 0.418 |  | 0.36 (0.17 - 0.73) | 0.005 |  | 0.58 (0.26 - 1.32) | 0.195 |
| Maintained |  | 1 (ref.) |  |  | 1 (ref.) |  |  | 1 (ref.) |  |
| Gained >5 kg |  | 0.69 (0.28 - 1.72) | 0.422 |  | 0.67 (0.28 - 1.60) | 0.364 |  | 0.75 (0.28 - 2.01) | 0.566 |
| ASV (kg) | 3.9 ± 0.3 | 4.7 ± 0.3 | 0.065 | 4.1 ± 0.3 | 4.3 ± 0.3 | 0.720 | 4.0 ± 0.4 | 4.3 ± 0.2 | 0.529 |
| VIM | 19.2 ± 1.3 | 22.1 ± 1.5 | 0.144 | 20.5 ± 1.5 | 20.3 ± 1.2 | 0.928 | 20.3 ± 1.9 | 20.4 ± 1.1 | 0.957 |
| **Waist data** |  |  |  |  |  |  |  |  |  |
| Waist (cm) | 106 ± 1 | 106 ± 1 | 0.801 | 106 ± 1 | 106 ± 1 | 0.918 | 107 ± 2 | 105 ± 1 | 0.559 |
| Abdominal obesity |  |  |  |  |  |  |  |  |  |
| Normal |  | 1 (ref.) |  |  | 1 (ref.) |  |  | 1 (ref.) |  |
| Obese |  | 1.08 (0.50 - 2.30) | 0.847 |  | 1.15 (0.55 - 2.38) | 0.716 |  | 0.95 (0.41 - 2.23) | 0.910 |
| Waist change (cm) | 2.6 ± 1.0 | 1.9 ± 1.1 | 0.615 | 2.0 ± 1.1 | 2.5 ± 0.9 | 0.749 | 1.8 ± 1.4 | 2.4 ± 0.8 | 0.713 |
| Waist change categories |  |  |  |  |  |  |  |  |  |
| Lost >5 cm |  | 3.17 (1.13 - 8.87) | 0.028 |  | 0.95 (0.37 - 2.43) | 0.909 |  | 1.29 (0.41 - 4.08) | 0.669 |
| Maintained |  | 1 (ref.) |  |  | 1 (ref.) |  |  | 1 (ref.) |  |
| Gained >5 cm |  | 1.20 (0.61 - 2.39) | 0.596 |  | 1.28 (0.66 - 2.51) | 0.462 |  | 1.05 (0.49 - 2.24) | 0.903 |
| ASV (cm) | 4.8 ± 0.3 | 5.4 ± 0.3 | 0.240 | 5.0 ± 0.4 | 5.1 ± 0.3 | 0.953 | 5.5 ± 0.4 | 4.9 ± 0.3 | 0.300 |
| VIM | 3.1 ± 0.2 | 3.5 ± 0.2 | 0.122 | 3.2 ± 0.2 | 3.3 ± 0.2 | 0.580 | 3.4 ± 0.3 | 3.2 ± 0.2 | 0.644 |

ASV, average successive variability of weight; BMI, body mass index; FPG, fasting plasma glucose; VIM, variability independent of the mean. Analysis was performed separately for each anthropometric variable (row), and models are not adjusted for the other row variables. Multivariable analysis for categorical variables was performed using logistic regression and results are expressed as odds ratio (95% confidence interval). Multivariable analysis for continuous variables was performed using analysis of variance and results are expressed as adjusted average ± standard error. Multivariable analysis adjusted for gender, age (continuous), s educational level (mandatory, apprenticeship, high school and university), marital status (alone/couple), smoking status (never, former, current), alcohol consumption (yes/no), presence of a diet (yes/no), antihypertensive drug treatment (yes/no) and hypolipidemic drug treatment (yes/no).

**Supplementary table 4**: bivariate analysis of weight parameters of the participants with type 2 diabetes mellitus, according to diabetes control status (yes/no) at the second follow-up (2014-2017) of the CoLaus study, Lausanne, Switzerland. Only participants who were treated for diabetes throughout the whole study period.

|  | **Using FPG levels (n=81)** | | | **HbA_1_c level <7.0% (n=80)** | | | **Age-dependent HbA_1_c levels (n=80)** | | |
| --- | --- | --- | --- | --- | --- | --- | --- | --- | --- |
|  | **No** | **Yes** | **p-value** | **No** | **Yes** | **p-value** | **No** | **Yes** | **p-value** |
| Number | 42 (51.8) | 39 (48.2) |  | 34 (42.5) | 46 (57.5) |  | 22 (25.0) | 58 (75.0) |  |
| **Body mass index data** |  |  |  |  |  |  |  |  |  |
| Body mass index (kg/m^2^) | 30.6 ± 4.7 | 29.7 ± 5.2 | 0.415 | 29.9 ± 4.7 | 30.1 ± 5.1 | 0.841 | 31.0 ± 4.5 | 29.7 ± 5.0 | 0.257 |
| BMI category (%) |  |  | 0.792 † |  |  | 0.983 |  |  | 0.325 † |
| Normal | 5 (11.9) | 7 (18.0) |  | 5 (14.7) | 7 (15.2) |  | 1 (4.6) | 11 (19.0) |  |
| Overweight | 17 (40.5) | 15 (38.5) |  | 14 (41.2) | 18 (39.1) |  | 10 (45.5) | 22 (37.9) |  |
| Obese | 20 (47.6) | 17 (43.6) |  | 15 (44.1) | 21 (45.7) |  | 11 (50.0) | 25 (43.1) |  |
| **Weight data** |  |  |  |  |  |  |  |  |  |
| Weight change (kg) | -0.5 ± 6.4 | -2.9 ± 9.5 | 0.186 | -1.2 ± 7.6 | -2.2 ± 8.3 | 0.600 | 0.1 ± 8.0 | -2.5 ± 8.0 | 0.205 |
| Weight change categories (%) |  |  | 0.390 † |  |  | 0.771 |  |  | 0.682 † |
| Lost >5 kg | 10 (23.8) | 13 (33.3) |  | 11 (32.4) | 12 (26.1) |  | 5 (22.7) | 18 (31.0) |  |
| Maintained | 27 (64.3) | 19 (48.7) |  | 18 (52.9) | 28 (60.9) |  | 13 (59.1) | 33 (56.9) |  |
| Gained >5 kg | 5 (11.9) | 7 (18.0) |  | 5 (14.7) | 6 (13.0) |  | 4 (18.2) | 7 (12.1) |  |
| ASV (kg) | 2.9 [2.0 - 4.4] | 4.1 [1.9 - 6.6] | 0.233 § | 3.4 [1.9 - 4.7] | 3.3 [2.0 - 5.9] | 0.964 § | 3.2 [1.9 - 4.5] | 3.4 [1.9 - 6.3] | 0.764 § |
| VIM | 13.7 [9.4-22.6] | 23.5 [10.1-36.2] | 0.061 § | 17.6 [9.4-24.9] | 14.0 [10.1-33.4] | 0.993 § | 15.1 [9.4-23.4] | 14.8 [10.1-33.8] | 0.605 § |
| **Waist data** |  |  |  |  |  |  |  |  |  |
| Waist (cm) | 109 ± 14 | 104 ± 14 | 0.157 | 107 ± 13 | 106 ± 14 | 0.829 | 110 ± 11 | 105 ± 14 | 0.103 |
| Abdominal obesity (%) | 33 (78.6) | 28 (73.7) | 0.608 | 25 (73.5) | 35 (77.8) | 0.662 | 17 (77.3) | 43 (75.4) | 1.000§ |
| Waist change (cm) | 3.2 ± 7.5 | -0.3 ± 15.2 | 0.187 | 3.0 ± 8.3 | 0.2 ± 14.1 | 0.311 | 3.8 ± 8.9 | 0.5 ± 12.9 | 0.285 |
| Waist change categories (%) |  |  | 0.127 † |  |  | 0.646 † |  |  | 0.286 † |
| Lost >5 cm | 3 (7.1) | 9 (23.7) |  | 4 (11.8) | 8 (17.8) |  | 1 (4.5) | 11 (19.3) |  |
| Maintained | 19 (45.2) | 14 (36.8) |  | 16 (47.1) | 17 (37.8) |  | 11 (50.0) | 22 (38.6) |  |
| Gained >5 cm | 20 (47.6) | 15 (39.5) |  | 14 (41.2) | 20 (44.4) |  | 10 (45.5) | 24 (42.1) |  |
| ASV (cm) | 3.5 [2.8 - 6.8] | 4.6 [2.9 - 8.3] | 0.383 § | 4.0 [2.5 - 7.5] | 4.0 [2.9 - 6.8] | 0.808 § | 5.1 [2.6 - 9.0] | 4.0 [2.8 - 6.8] | 0.484 § |
| VIM | 2.5 [1.9 - 4.6] | 3.4 [2.1 - 4.6] | 0.316 § | 2.9 [1.8 - 4.9] | 2.4 [2.1 - 4.4] | 0.913 § | 3.3 [1.8 - 5.7] | 2.7 [2.1 - 4.4] | 0.702 § |

ASV, average successive variability of weight; BMI, body mass index; FPG, fasting plasma glucose; VIM, variability independent of the mean. Results are expressed as number of participants (percentage) for categorical variables and as average ± standard deviation or as median [interquartile range] for continuous variables. Bivariate analysis performed using chi-square or Fisher’s exact test (†) for categorical variables and student’s t-test or Kruskal-Wallis test (§) for continuous variables.

**Supplementary table 5**: multivariable analysis of weight parameters of the participants with type 2 diabetes mellitus, according to diabetes control status (yes/no) at the second follow-up (2014-2017) of the CoLaus study, Lausanne, Switzerland. Only participants who were treated for diabetes throughout the whole study period.

|  | **Using FPG levels (n=81)** | | | **HbA_1_c level <7.0% (n=80)** | | | **Age-dependent HbA_1_c levels (n=80)** | | |
| --- | --- | --- | --- | --- | --- | --- | --- | --- | --- |
|  | **No** | **Yes** | **p-value** | **No** | **Yes** | **p-value** | **No** | **Yes** | **p-value** |
| Number | 42 (51.8) | 39 (48.2) |  | 34 (42.5) | 46 (57.5) |  | 22 (25.0) | 58 (75.0) |  |
| **Body mass index data** |  |  |  |  |  |  |  |  |  |
| Body mass index (kg/m^2^) | 30.4 ± 0.8 | 29.9 ± 0.8 | 0.671 | 30.0 ± 0.8 | 30.1 ± 0.7 | 0.919 | 30.8 ± 1.0 | 29.7 ± 0.6 | 0.413 |
| BMI category |  |  |  |  |  |  |  |  |  |
| Normal |  | 1 (ref.) |  |  | 1 (ref.) |  |  | 1 (ref.) |  |
| Overweight |  | 0.22 (0.04 - 1.27) | 0.090 |  | 0.59 (0.12 - 2.91) | 0.521 |  | 0.10 (0.01 - 1.30) | 0.078 |
| Obese |  | 0.32 (0.05 - 1.85) | 0.202 |  | 0.69 (0.14 - 3.44) | 0.652 |  | 0.1.0 (0.01 - 1.42) | 0.089 |
| **Weight data** |  |  |  |  |  |  |  |  |  |
| Weight change (kg) | -1.0 ± 1.2 | -2.4 ± 1.2 | 0.438 | -0.8 ± 1.3 | -2.5 ± 1.1 | 0.360 | -0.3 ± 1.7 | -2.4 ± 1.0 | 0.302 |
| Weight change categories |  |  |  |  |  |  |  |  |  |
| Lost >5 kg |  | 2.00 (0.53 - 7.50) | 0.304 |  | 0.70 (0.19 - 2.50) | 0.579 |  | 1.19 (0.25 - 5.53) | 0.827 |
| Maintained |  | 1 (ref.) |  |  | 1 (ref.) |  |  | 1 (ref.) |  |
| Gained >5 kg |  | 5.57 (0.97 - 31.9) | 0.054 |  | 0.81 (0.16 - 3.97) | 0.793 |  | 0.70 (0.12 - 4.19) | 0.697 |
| ASV (kg) | 3.5 ± 0.5 | 5.1 ± 0.5 | 0.040 | 4.2 ± 0.5 | 4.2 ± 0.5 | 0.977 | 4.1 ± 0.7 | 4.2 ± 0.4 | 0.838 |
| VIM | 16.0 ± 2.1 | 26.0 ± 2.2 | 0.002 | 19.5 ± 2.4 | 21.6 ± 2.1 | 0.541 | 19.2 ± 3.1 | 21.3 ± 1.8 | 0.588 |
| **Waist data** |  |  |  |  |  |  |  |  |  |
| Waist (cm) | 109 ± 2 | 105 ± 2 | 0.214 | 107 ± 2 | 106 ± 2 | 0.654 | 110 ± 3 | 105 ± 2 | 0.176 |
| Abdominal obesity |  |  |  |  |  |  |  |  |  |
| Normal |  | 1 (ref.) |  |  | 1 (ref.) |  |  | 1 (ref.) |  |
| Obese |  | 0.35 (0.09 - 1.43) | 0.145 |  | 1.05 (0.28 - 3.92) | 0.945 |  | 0.78 (0.15 - 3.89) | 0.758 |
| Waist change (cm) | 2.4 ± 1.9 | 0.6 ± 2.0 | 0.539 | 3.6 ± 2.1 | -0.2 ± 1.8 | 0.186 | 4.3 ± 2.7 | 0.3 ± 1.6 | 0.219 |
| Waist change categories |  |  |  |  |  |  |  |  |  |
| Lost >5 cm |  | 7.25 (1.17 - 44.8) | 0.033 |  | 2.52 (0.50 - 12.8) | 0.265 |  | 6.38 (0.58 - 70.5) | 0.130 |
| Maintained |  | 1 (ref.) |  |  | 1 (ref.) |  |  | 1 (ref.) |  |
| Gained >5 cm |  | 1.44 (0.45 - 4.68) | 0.539 |  | 0.97 (0.32 - 2.97) | 0.958 |  | 0.97 (0.27 - 3.44) | 0.959 |
| ASV (cm) | 4.7 ± 0.5 | 5.9 ± 0.6 | 0.184 | 5.2 ± 0.6 | 5.2 ± 0.5 | 0.988 | 6.2 ± 0.8 | 4.9 ± 0.5 | 0.123 |
| VIM | 3.0 ± 0.3 | 3.8 ± 0.3 | 0.137 | 3.4 ± 0.4 | 3.4 ± 0.3 | 0.946 | 3.8 ± 0.5 | 3.2 ± 0.3 | 0.290 |

ASV, average successive variability of weight; BMI, body mass index; FPG, fasting plasma glucose; VIM, variability independent of the mean. Analysis was performed separately for each anthropometric variable (row), and models are not adjusted for the other row variables. Multivariable analysis for categorical variables was performed using logistic regression and results are expressed as odds ratio (95% confidence interval). Multivariable analysis for continuous variables was performed using analysis of variance and results are expressed as adjusted average ± standard error. Multivariable analysis adjusted for gender, age (continuous), educational level (mandatory, apprenticeship, high school and university), marital status (alone/couple), smoking status (never, former, current), alcohol consumption (yes/no), presence of a diet (yes/no), antihypertensive drug treatment (yes/no) and hypolipidemic drug treatment (yes/no).

**Supplementary table 6**: multivariable analysis of weight parameters of the participants with type 2 diabetes mellitus, according to diabetes control status (yes/no) at the second follow-up (2014-2017) of the CoLaus study, Lausanne, Switzerland. With further adjustment on antidiabetic drug categories.

|  | **Using FPG levels (n=267)** | | | **HbA_1_c level <7.0% (n=266)** | | | **Age-dependent HbA_1_c levels (n=266)** | | |
| --- | --- | --- | --- | --- | --- | --- | --- | --- | --- |
|  | **No** | **Yes** | **p-value** | **No** | **Yes** | **p-value** | **No** | **Yes** | **p-value** |
| Number | 130 (48.7) | 137 (51.3) |  | 94 (35.3) | 172 (64.7) |  | 69 (26.0) | 197 (74.0) |  |
| **Body mass index data** |  |  |  |  |  |  |  |  |  |
| Body mass index (kg/m^2^) | 30.0 ± 0.4 | 30.6 ± 0.4 | 0.341 | 30.0 ± 0.5 | 30.4 ± 0.4 | 0.533 | 30.4 ± 0.6 | 30.3 ± 0.3 | 0.872 |
| BMI category |  |  |  |  |  |  |  |  |  |
| Normal |  | 1 (ref.) |  |  | 1 (ref.) |  |  | 1 (ref.) |  |
| Overweight |  | 0.89 (0.38 - 2.07) | 0.783 |  | 1.01 (0.41 - 2.50) | 0.982 |  | 0.72 (0.23 - 2.24) | 0.575 |
| Obese |  | 1.09 (0.46 - 2.57) | 0.847 |  | 0.99 (0.40 - 2.46) | 0.976 |  | 0.61 (0.19 - 1.89) | 0.388 |
| **Weight data** |  |  |  |  |  |  |  |  |  |
| Weight change (kg) | -0.4 ± 0.7 | 0.6 ± 0.7 | 0.293 | -1.6 ± 0.8 | 1.0 ± 0.6 | 0.014 | -2.0 ± 1.0 | 0.8 ± 0.6 | 0.020 |
| Weight change categories |  |  |  |  |  |  |  |  |  |
| Lost >5 kg |  | 0.70 (0.36 - 1.34) | 0.276 |  | 0.24 (0.12 - 0.50) | <0.001 |  | 0.4 (0.18 - 0.89) | 0.025 |
| Maintained |  | 1 (ref.) |  |  | 1 (ref.) |  |  | 1 (ref.) |  |
| Gained >5 kg |  | 1.42 (0.73 - 2.76) | 0.303 |  | 1.16 (0.55 - 2.48) | 0.695 |  | 1.63 (0.69 - 3.85) | 0.266 |
| ASV (kg) | 3.9 ± 0.3 | 4.8 ± 0.3 | 0.033 | 4.2 ± 0.3 | 4.4 ± 0.2 | 0.601 | 4.1 ± 0.4 | 4.4 ± 0.2 | 0.540 |
| VIM | 19.0 ± 1.3 | 23.9 ± 1.3 | 0.011 | 20.9 ± 1.6 | 21.7 ± 1.2 | 0.695 | 20.1 ± 2.0 | 21.9 ± 1.1 | 0.460 |
| **Waist data** |  |  |  |  |  |  |  |  |  |
| Waist (cm) | 105 ± 1 | 106 ± 1 | 0.419 | 105 ± 1 | 106 ± 1 | 0.759 | 106 ± 1 | 105 ± 1 | 0.590 |
| Abdominal obesity |  |  |  |  |  |  |  |  |  |
| Normal |  | 1 (ref.) |  |  | 1 (ref.) |  |  | 1 (ref.) |  |
| Obese |  | 1.11 (0.59 - 2.08) | 0.757 |  | 1.02 (0.52 - 2.01) | 0.956 |  | 0.80 (0.36 - 1.78) | 0.590 |
| Waist change (cm) | 2.9 ± 0.8 | 3.9 ± 0.8 | 0.401 | 2.1 ± 1.0 | 4.1 ± 0.7 | 0.143 | 1.7 ± 1.2 | 4.0 ± 0.7 | 0.126 |
| Waist change categories |  |  |  |  |  |  |  |  |  |
| Lost >5 cm |  | 3.22 (1.26 - 8.18) | 0.014 |  | 0.98 (0.39 - 2.43) | 0.963 |  | 1.19 (0.40 - 3.48) | 0.755 |
| Maintained |  | 1 (ref.) |  |  | 1 (ref.) |  |  | 1 (ref.) |  |
| Gained >5 cm |  | 1.76 (1.00 - 3.09) | 0.051 |  | 1.79 (0.97 - 3.31) | 0.065 |  | 1.55 (0.78 - 3.11) | 0.214 |
| ASV (cm) | 4.8 ± 0.3 | 6.1 ± 0.3 | 0.002 | 5.1 ± 0.4 | 5.6 ± 0.3 | 0.267 | 5.4 ± 0.4 | 5.4 ± 0.2 | 0.910 |
| VIM | 3.0 ± 0.2 | 4.1 ± 0.2 | <0.001 | 3.2 ± 0.2 | 3.8 ± 0.2 | 0.043 | 3.2 ± 0.3 | 3.7 ± 0.2 | 0.212 |

ASV, average successive variability of weight; BMI, body mass index; FPG, fasting plasma glucose; VIM, variability independent of the mean. Analysis for categorical variables was performed using logistic regression and results are expressed as odds ratio (95% confidence interval). Analysis for continuous variables was performed using analysis of variance and results are expressed as adjusted average ± standard error. Analysis adjusted for gender, age (continuous), smoking status (never, former, current), presence of a diet (yes/no), educational level (mandatory, apprenticeship, high school and university), alcohol consumption (yes/no) and antidiabetic drug categories: biguanides (yes/no), insulin (yes/no), dipeptidylpeptidase-4 inhibitors (yes/no), glucagon-like peptide-1 analogues (yes/no), sodium-glucose co-transporter-2 inhibitors (yes/no) and other antidiabetics (yes/no).

**Supplementary figure 1**: distribution of glycemic control categories by weight change categories. Only participants who were treated for diabetes throughout the whole study period. Results are expressed as number of participants (upper panel) or percentage (lower panel).


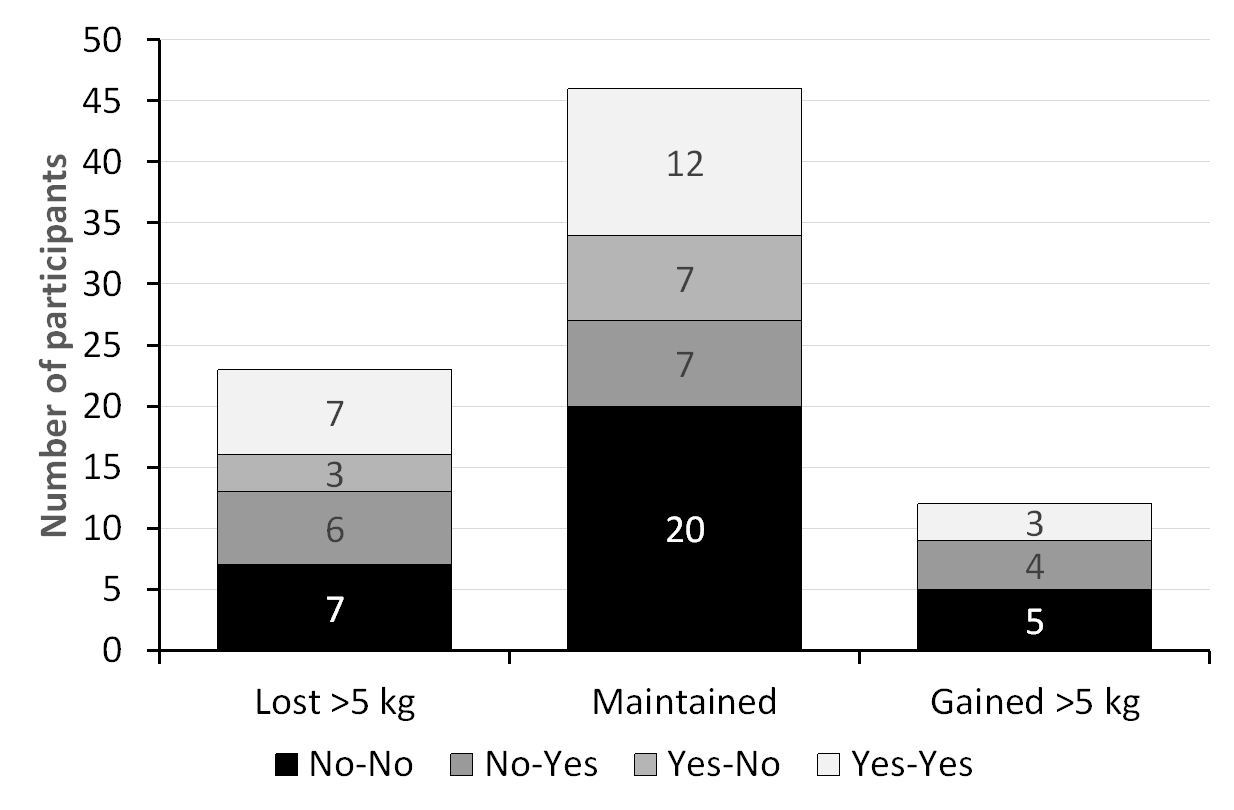


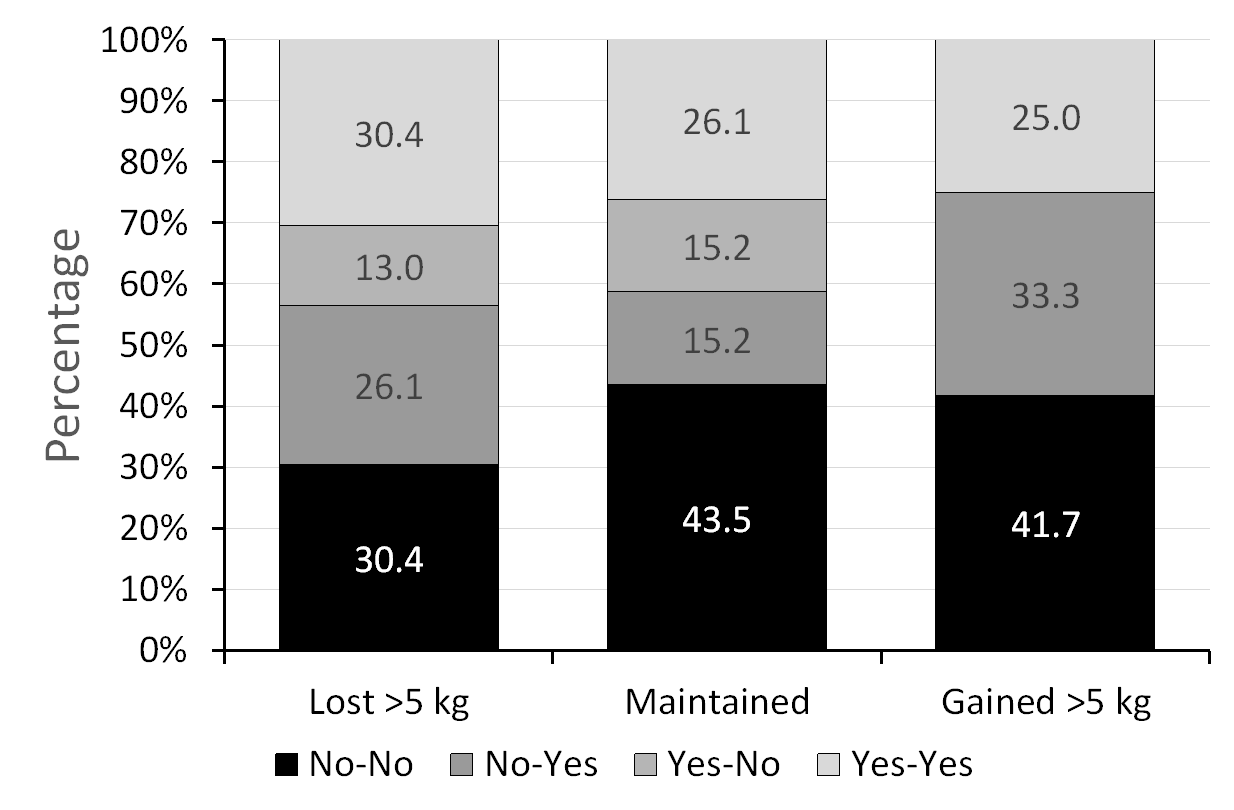

Supplement: Supplementary data 1 [file mmc1.docx]
